# Pharmacologic Treatment of Malignant Catatonia Without Electroconvulsive Therapy: A Case Report

**DOI:** 10.7759/cureus.58071

**Published:** 2024-04-11

**Authors:** Conor Hegewald, Danielle Guthrie, Sydney M LeFay

**Affiliations:** 1 Psychiatry, Samaritan Health Services, Corvallis, USA; 2 Psychiatry, District Medical Group, Phoenix, USA; 3 Psychiatry, Salem Health, Salem, USA; 4 Psychiatry, Oregon Health & Science University, Portland, USA

**Keywords:** consult-liaison psychiatry, electroconvulsive therapy (ect), bipolar i disorder, lethal catatonia, malignant catatonia

## Abstract

Malignant catatonia is a rare, life-threatening variant of catatonia requiring prompt treatment. Malignant catatonia is characterized by typical catatonia symptoms of psychomotor, neurologic, and behavioral changes complicated by autonomic instability, with an estimated mortality rate of 50% or more when untreated. Electroconvulsive therapy (ECT) is considered the definitive and most effective treatment for malignant catatonia, with minimal literature on the efficacy of pharmacological interventions alone. Timely access to life-saving ECT may be limited in some hospitals due to restrictive laws on the use of ECT when the patient is incapacitated or due to lack of treatment availability. This case report describes the successful pharmacologic treatment of a patient with malignant catatonia where ECT was unobtainable due to legal restrictions and lack of access to treatment. The patient was initially commenced on lorazepam but continued to deteriorate, subsequently developing complications of aspiration pneumonia and *Clostridium difficile* colitis. The patient's malignant catatonia resolved with a combination of lorazepam, memantine, and a one-time dose of dantrolene. This complex case highlights the challenges of treating malignant catatonia in under-resourced systems or jurisdictions with restrictive ECT laws and adds additional data on the successful use of pharmacologic interventions for malignant catatonia where ECT is impractical or delayed.

## Introduction

Catatonia is a complex neuropsychiatric condition with disturbances in behavioral and motor activity, defined in the Diagnostic and Statistical Manual of Mental Disorders (DSM), 5th edition, as exhibiting three or more symptoms of the following: stupor, catalepsy, waxy flexibility, mutism, negativism, posturing, mannerism, stereotypy, agitation, grimacing, echolalia, and echopraxia [1]. Malignant catatonia, also known as lethal catatonia, is the most severe of the catatonia subtypes and is characterized by typical catatonia symptoms, with additional findings of dysautonomia (such as hypertension or tachycardia) and fever. If left untreated, malignant catatonia is thought to have a mortality rate of approximately 50% [2,3]. Common laboratory findings in malignant catatonia are nonspecific but may include elevated creatinine kinase (CK), elevated white blood cells, and low serum iron [2]. Recognition and prompt intervention for catatonia with malignant features are crucial to reducing rates of morbidity and mortality [4].

Catatonia has a variety of psychiatric and non-psychiatric causes. Primary psychiatric disorders causing catatonia include bipolar disorder, major depressive disorder, or schizophrenia; medical causes of catatonia may include autoimmune conditions, electrolyte abnormalities, structural neurologic disorders, or medication-induced etiologies such as the well-known clozapine withdrawal catatonia [5,6]. Neuroleptic malignant syndrome (NMS) has been called by some researchers a form of “drug-induced” catatonia, with the two presenting so similarly that they can be nearly indistinguishable, especially when the patient is prescribed antipsychotics [7]. These two disorders are thought to be on the same spectrum with similar pathophysiology; while the pathophysiology is not fully understood, this is postulated to involve disturbances of GABAergic and dopaminergic systems [8]. More recently, the glutamatergic system has been implicated in the pathophysiology of catatonia, with evidence of efficacy for the use of N-methyl-D-aspartate (NMDA) antagonist agents in the treatment of catatonia and the emergence of catatonia in the setting of anti-NMDA-receptor encephalitis [9,10].

Existing literature is limited regarding the treatment of malignant catatonia without electroconvulsive therapy (ECT). ECT is widely accepted by experts as a first-line therapy for malignant catatonia, often in conjunction with intravenous lorazepam [11]. Benzodiazepines, classically used as intravenous lorazepam, have a long and established history as the first-line treatment for non-malignant catatonia [2]. In a systematic review by Beach et al., an algorithm was proposed for alternative treatments for catatonia in the setting of limited access to ECT, with the caveat that ECT remains the definitive treatment for catatonia, particularly malignant catatonia [11]. This algorithm recommends beginning with intravenous lorazepam, then continuing with trials of NMDA antagonist agents such as memantine or amantadine, antiepileptics such as valproic acid or carbamazepine, and finally antipsychotic agents such as olanzapine, clozapine, or aripiprazole in combination with lorazepam [11]. The 2023 British Association for Psychopharmacology consensus guidelines on the management of catatonia similarly recommend adjunctive use of NMDA antagonist agents if lorazepam monotherapy fails and ECT either fails or is unavailable, again emphasizing the importance of prompt use of ECT within 48-72 hours for catatonia with malignant features [12]. Some literature has referenced the use of bromocriptine, a semisynthetic ergot alkaloid derivative and a sympatholytic dopamine D2 receptor agonist, or dantrolene, a postsynaptic muscle relaxant that interferes with the release of calcium ions from the sarcoplasmic reticulum, in treatment-resistant cases of catatonia, given their utility in the treatment of NMS and the likely shared pathophysiology with malignant catatonia [13].

The scarce literature on successfully treating malignant catatonia without ECT lends to uncertainty in regions where access to ECT is limited or where consent cannot be obtained [11,14]. Here, we describe a case in Oregon, USA, where a patient presented to the hospital with malignant catatonia and was successfully treated with pharmacotherapy alone due to the legal and practical limitations of obtaining emergent ECT.

## Case presentation

A 64-year-old female with a past psychiatric history of bipolar I disorder and catatonia, and other medical history of hypertension, chronic kidney disease, dyslipidemia, and coronary artery disease, presented to the emergency department for worsening confusion over the prior two weeks. The patient’s bipolar disorder had been previously managed with a combination of valproic acid 750 mg twice daily and haloperidol 2 mg daily. The patient had discontinued quetiapine 300 mg nightly within the month before presentation due to sedation.

Initial physical examination by the hospitalist service in the emergency department was notable for mutism, negativism, stupor, posturing, rigidity, and stereotypy (see Table 1 for catatonia symptom definitions). Blood pressure was elevated at 169/95, but other vital signs were unremarkable. Laboratory workup revealed no findings indicative of infection, metabolic abnormalities, or drug toxicity. Serum valproic acid level was found to be 104.7 mcg/mL. The Bush-Francis Catatonia Rating Scale (BFCRS), calculated due to high suspicion for hypokinetic catatonia, was scored at nine. Haloperidol and valproic acid were discontinued. The hospitalist service initiated lorazepam 2 mg three times daily, and the patient was admitted to the medical floor for further evaluation and treatment.

**Table 1:** DSM-5 catatonia symptoms and descriptions This table reflects symptoms and definitions as described in the chapter *Schizophrenia Spectrum and Other Psychotic Disorders *from the *Diagnostic and Statistical Manual of Mental Disorders,* *5th edition* (text revision). American Psychiatric Association Publishing, 2022. DSM-5: Diagnostic and Statistical Manual of Mental Disorders, 5th edition.

Symptom	Definition
Stupor	Lack of psychomotor activity; no interaction with the external environment
Catalepsy	Passively holding a position against gravity or posture remains fixed regardless of external forces
Waxy flexibility	Gentle resistance to external/examiner positioning; limbs may be manipulated into position by an examiner and passively held by the subject without prompting
Mutism	Reduced or absent verbal responses
Negativism	Oppositional or absent responses to examiner instructions or questions
Posturing	Fixed maintenance of a spontaneously held posture against gravity
Mannerism	Unusual or purposeless motions may be seen as odd exaggerations of normal motor activities
Stereotypy	Repeated purposeless motions without a clear goal
Agitation	Agitated or restless behaviors not impacted by external cues
Grimacing	Observed grimacing facial expression
Echolalia	Echoing or repetition of another's verbal expressions
Echopraxia	Echoing or repetition of another's movements

After 24 hours, the hospitalist service became concerned that lorazepam was increasing the patient’s sedation and reduced lorazepam to 0.5 mg three times daily. Twenty-four hours after the reduction of the dose, the patient’s mental status and physical condition worsened. The psychiatry service was consulted on hospital day four, where she was found to be tremulous, diffusely rigid, and unable to participate in the interview. Vital signs were notable for fever (39°C), tachycardia (139 beats/min), and worsening hypertension (210/104). Laboratory studies revealed leukocytosis (15.45 x 10^9/L) and elevated creatine kinase (2300 U/L).

Further laboratory and diagnostic testing, including brain MRI, to assess potential medical etiologies of catatonia such as CNS infection (meningitis versus encephalitis), systemic infections, hydrocephalus, autoimmune pathology, and paraneoplastic pathology were negative. The etiology of the patient's catatonia was suspected to be due to a primary psychiatric cause given her prior history of bipolar disorder, with no objective evidence of other medical causes. Physical examination, vitals, and laboratory testing supported a diagnosis of malignant catatonia, with a rule-out diagnosis of NMS given the recent use of haloperidol.

Intravenous lorazepam was increased to 4 mg four times daily (16 mg per 24 hours) with minimal improvement on day four. Acknowledging that the definitive treatment for malignant catatonia is ECT, the treatment team began investigating local treatment facilities with ECT availability. Due to the patient’s mutism and rapidly worsening medical status, she did not have the capacity to consent to ECT. According to Oregon law, a person without the capacity to consent must be under guardianship with a special ECT clause or imminently likely to perish of their condition to receive ECT; even if these criteria were met, the team was advised that an ethics consultation would likely be required by the receiving hospital, if one could be found. While two Oregon hospital systems with associated ECT services were identified, neither was able to accept the patient as they did not have regular privileges for the use of ECT in a medical or intensive care setting.

On hospital day five, the patient remained acutely ill with a BFCRS score of 21, so intravenous lorazepam was increased further to 5 mg four times daily (20 mg per 24 hours). After a discussion with the patient’s next of kin, they agreed with a transfer to ECT out of state if available, as well as a trial of dantrolene due to persistent rigidity and hyperthermia, and the rule-out diagnosis of NMS. A single dose of intravenous dantrolene 1 mg/kg was administered. A search for ECT performed in a medical setting in neighboring states was started; however, that evening the patient developed severe respiratory insufficiency secondary to aspiration pneumonia.

On hospital day six, lorazepam treatment was held by the intensive care team due to concern for worsening respiratory depression. The patient developed diarrhea and tested positive for *Clostridium difficile* colitis, for which she was started on vancomycin via placement of a nasogastric (NG) tube. Despite these medical complications, her autonomic instability (blood pressure, temperature, heart rate) showed signs of improvement. The addition of an NG tube allowed for the trial of memantine starting at 5 mg daily as an additional adjunctive treatment. Lorazepam was reinstated at 2 mg three times daily after extensive conversation with the intensive care team and the patient’s family regarding the risks of worsened catatonia weighed alongside the risks of respiratory depression.

Following the initiation of memantine and restarting lorazepam, the BFCRS score decreased to 14. Over the coming days, the patient continued to show physical and diagnostic signs of improvement and was able to transfer from the ICU to the medical floor on hospital day 10.

As the patient improved, she was able to participate in an interview. Although she never endorsed any mood or psychotic symptoms while hospitalized, she was resumed on valproic acid 500 mg twice daily on day 18 of her hospitalization given her long history of bipolar I disorder and the utility of antiepileptics in the treatment of catatonia. She was not restarted on antipsychotic treatment while hospitalized due to concerns for worsening catatonia and a lack of objective signs of psychosis. Lorazepam was progressively reduced to 0.5 mg three times daily, while memantine was increased to 10 mg twice daily (20 mg daily) before discharge on day 21 of her hospitalization. The patient was seen at a primary care appointment six days following discharge, where she appeared to be back to her baseline level of function, with no evidence of ongoing catatonic symptoms.

## Discussion

ECT demonstrates the best evidence for the reduction in mortality rates and resolution of symptoms in malignant catatonia [2,4,12]. In the case described here, legal barriers and poor treatment availability significantly delayed treatment such that the patient was too unstable to transfer, leading to additional medication trials and complications. Fortunately, adjunctive alternative medication trials were successful for this patient. The inability to access the most effective treatment for this patient’s life-threatening condition was distressing to the treatment team members and the patient’s family, all of whom supported the use of ECT, and the patient developed aspiration pneumonia, a known complication of catatonia [6,15].

Laws regarding ECT may not offer provisions for exceptions in medically unstable patients, as was the case for this patient [16]. Approximately 20% of catatonic presentations are due to a general medical condition, and in some jurisdictions, mental health involuntary treatment laws pertaining to ECT may not apply to patients for whom the primary reason for treatment is medical [16,17].

For medically unstable patients, regardless of their legal status, many regions in the United States and throughout the world may lack the availability of ECT practitioners, facilities, or privileges to offer those services in an ICU or medical floor setting [14]. Patients in hospitals lacking ECT equipment and staff are left seeking transfers to another facility, potentially further complicating their legal status if transferring to a region with different laws, and ultimately further delaying access to life-saving treatment [4,11,16].

For clinicians treating malignant catatonia without ECT access, limited research is available to indicate which pharmacologic treatments are most efficacious in malignant catatonia. Given the ethical complications of researching a less-effective treatment for a life-threatening condition with an existing, effective treatment already available, additional case reports on malignant catatonia treated with medications alone and analysis of combined case studies may be the most appropriate means to support the decision-making of clinicians with a lack of access or legal barriers to ECT. While there is limited data available on adjunctive catatonia treatments, there is some evidence for the use of NMDA antagonist medications (such as memantine) [11,13]. Adjunctive treatment with dantrolene may be of use due to the shared pathophysiology between NMS and malignant catatonia, with greater consideration in cases where NMS is part of the differential diagnosis, as dantrolene may reduce mortality in cases of NMS [12]. In this case, while malignant catatonia seems the most likely diagnosis due to the worsening of catatonic symptoms following discontinuation of antipsychotics, NMS may still have played a role in her presentation and influenced the psychiatry team's treatment decisions. Multiple adjunctive approaches (memantine and dantrolene) were required in addition to lorazepam due to the lack of access to ECT as a definitive treatment.

## Conclusions

Malignant catatonia is a life-threatening condition requiring prompt recognition and treatment. Barriers to accessing ECT, including lack of local services and regionally-dictated legal complications, may delay treatment. Delayed treatment of malignant catatonia may lead to worsened morbidity and mortality, and poorer overall treatment responses in catatonia. In the case presented here, the combined use of dantrolene, memantine, and intravenous lorazepam effectively treated the patient’s malignant catatonia in the absence of access to ECT. Lorazepam, with or without adjunctive medications, may be the best management choice when treatment without ECT is necessary. Although pharmacologic interventions may prove successful in some cases of malignant catatonia, available literature continues to emphasize the prompt use of ECT as the most efficacious intervention. Urgent systems-level action is needed to ensure equitable access to evidence-based, life-saving medical care for patients with malignant catatonia and related conditions.
